# A Mixed-Methods Protocol to Identify Best Practices for Implementing Pharmacogenetic Testing in Clinical Settings

**DOI:** 10.3390/jpm12081313

**Published:** 2022-08-13

**Authors:** Nina R. Sperber, Deborah Cragun, Megan C. Roberts, Lisa M. Bendz, Parker Ince, Sarah Gonzales, Susanne B. Haga, R. Ryanne Wu, Natasha J. Petry, Laura Ramsey, Ryley Uber

**Affiliations:** 1 Department of Population Health Sciences, School of Medicine, Duke University, Durham, NC 27701, USA; 2 Durham VA Health Care System, Durham, NC 27705, USA; 3 College of Public Health, University of South Florida, Tampa, FL 33612, USA; 4 UNC Eshelman School of Pharmacy, University of North Carolina–Chapel Hill, Chapel Hill, NC 27599, USA; 5 Center for Medication Policy and Drug Information, Department of Pharmacy, Duke University Hospital, Durham, NC 27710, USA; 6 Department of Medicine, Duke University, Durham, NC 27701, USA; 7 School of Pharmacy, North Dakota State University/Sanford Health Imagenetics, Fargo, ND 58108, USA; 8 Department of Pediatrics, Divisions of Clinical Pharmacology and Research in Patient Services, University of Cincinnati College of Medicine, Cincinnati Children’s Hospital Medical Center, Cincinnati, OH 45229, USA; 9 Center for Pharmacy Innovation and Outcomes, Geisinger, Danville, CA 17822, USA

**Keywords:** pharmacogenomics, coincidence analysis, configurational comparative methods, health services research, implementation science, mixed methods

## Abstract

Using a patient’s genetic information to inform medication prescriptions can be clinically effective; however, the practice has not been widely implemented. Health systems need guidance on how to engage with providers to improve pharmacogenetic test utilization. Approaches from the field of implementation science may shed light on the complex factors affecting pharmacogenetic test use in real-world settings and areas to target to improve utilization. This paper presents an approach to studying the application of precision medicine that utilizes mixed qualitative and quantitative methods and implementation science frameworks to understand which factors or combinations consistently account for high versus low utilization of pharmocogenetic testing. This approach involves two phases: (1) collection of qualitative and quantitative data from providers—the cases—at four clinical institutions about their experiences with, and utilization of, pharmacogenetic testing to identify salient factors; and (2) analysis using a Configurational Comparative Method (CCM), using a mathematical algorithm to identify the minimally necessary and sufficient factors that distinguish providers who have higher utilization from those with low utilization. Advantages of this approach are that it can be used for small to moderate sample sizes, and it accounts for conditions found in real-world settings by demonstrating how they coincide to affect utilization.

## 1. Introduction

Use of genetic information to inform prescribing decisions (genotype-guided prescribing) is clinically effective and beneficial for many medications, yet not routinely implemented [1,2,3,4,5]. To promote appropriate utilization and greater adoption, the international evidence-based guideline organization, Clinical Pharmacogenetics Implementation Consortium (CPIC), has developed clinical recommendations for 46 gene-drug pairs that have high or moderate evidence [6]. Many of the medications commonly prescribed in the US have a CPIC guideline and have a high risk of either an adverse drug reaction or poor clinical response in the setting of certain genetic variants [1,2]. It is estimated that medications with CPIC guidance comprise about 18% of the 4 billion outpatient prescriptions in the US [5]. In addition, in the few settings where genotype-guided prescribing is part of routine clinical care, 99% of those tested have actionable variants that affect prescribing of at least one medication [3,4,7,8,9,10,11]. For these medications, pharmacogenetic test results could guide medication choice or dose, thus optimizing outcomes [12]. For example, enzymes encoded by the CYP2C19 and CYP2D6 genes impact how individuals metabolize certain antidepressants. According to CPIC guidelines, due to genetic variation, CYP2C19 poor metabolizers should initiate citalopram or escitalopram at 50% of the starting dose due to increased risk of side effects and CYP2D6 ultrarapid metabolizers should avoid paroxetine therapy due to increased probability of therapeutic failure [13]. Thus, testing for these genes could reduce trial and error that would occur without this additional information.

In addition to the CPIC guidelines, evidence-based guidance about how to implement genotype-guided prescribing into routine care in real-world settings is needed. To date, genotype-guided prescribing still largely occurs in research settings. Several papers have listed barriers and facilitators from providers’ perspectives, identified via surveys or qualitative methods, for the use of genetic testing for prescribing. Barriers to implementation have included uncertainty about: advantages of pharmacogenetics over their current practice, their ability to interpret the information and thus explain to patients and make clinical decisions, how to incorporate testing into current workflows, and cost and reimbursement implications [4,8,14]. Facilitators include provider training and education conducted by a pharmacist, including opportunity to learn by testing themselves, and point of care clinical decision support, which have boosted providers’ self-reported acceptance of and comfort with using genetic information when prescribing medication [4,8]. Additionally, progress has been made with respect to the key barrier of reimbursement and coverage in that United Healthcare and the Centers for Medicare and Medicaid Services (CMS) recently started covering genotype-guided prescribing for antidepressants according to CPIC guideline-based gene-drug pairs, underscoring greater acceptance by payors of genotype-guided therapy [15,16]. However, while greater coverage constitutes one step toward facilitating widespread use, it does not necessarily equate with uptake by providers during routine care [17]. To improve uptake, pharmacogenetic program leaders need evidence-based guidance that accounts for the complexity of everyday practice, namely how different aspects of individual behavior and organizational context intersect to affect utilization [18]. While prior work has described providers’ reports of factors important for their use of pharmacogenetics, these studies lack any evaluation of interdependence between these factors and their causal relationship to test utilization [4,8,14,17,18].

Configurational Comparative Methods (CCMs) can contribute toward the study of pharmacogenetics implementation by accounting for both complexity of and interdependencies between factors in small to moderate sample sizes [19]. CCMs employ mathematical algorithms to identify necessary and sufficient conditions for a desired outcome across a set of cases (e.g., providers). A recent addition to the CCM family, Coincidence Analysis (CNA), uses an algorithm that can reveal causal pathways (if substantiated by the data). Causal pathways occur when one or more conditions lead to another intermediate outcome, which then leads to the final outcome. Often utilized in sociological and public policy research in the past, health services researchers have increasingly added this methodology to their toolbox. Since 2005, approximately 28 health services-related publications indexed in PubMed have used CCMs, though only two have dealt with genomic/precision medicine and implementation science. Cragun et al. (2014) studied implementation processes that distinguished institutions with higher rates of universal tumor screening for Lynch syndrome and found that high performing institutions had a common set of implementation conditions, specifically targeted reflexive testing processes, and genetic counselor disclosure of positive test results with no barriers to contacting patients or obtaining a genetic counseling referral [20]. Rahm et al. (2018) described a protocol to use CCM to understand the variability of genomic/precision screening in colorectal cancer patients in multiple healthcare systems [21]. In this paper, we introduce this methodological approach to the study of pharmacogenetics testing uptake.

## 2. Materials and Methods

To understand which factors or combinations thereof consistently make a difference for high versus low utilization of pharmocogenetic testing, we designed a case-based approach that utilizes mixed qualitative and quantitative methods grounded in implementation science frameworks. The overall approach involves collection of qualitative and quantitative data from providers—the cases—at four clinical institutions about their experiences with pharmacogenetic testing and then application of CNA to demonstrate through mathematical modeling the minimally necessary and sufficient factors that distinguish cases based on their level of pharmacogenetics utilization. We draw from taxonomies developed in the field of implementation science, the study of methods to advance uptake of evidence-based practices, to define individual and organizational level factors. CCMs offer a way to observe how these factors manifest in conjunction with each other and contribute to the outcome of interest, high pharmacogenetic test utilization, using data from real-world experiences.

### 2.1. Cases

Cases consist of *providers who prescribe antidepressant medications* within the context of four clinical institutions that vary in use of pharmacogenetic testing. We aim to purposefully sample eight providers from each of the four institutions, with a goal of recruiting four high users and four low users and, among those, a mix of primary care and psychiatric providers, for a total of 32 cases; recruitment will occur until the sampling quotas from each site are met. We define “user” as a provider who either orders a test for CYP2C19 and CYP2D6 or uses/discloses results of a test automatically generated by their clinical institution [6]. Contributors from each clinical institution will provide a sampling frame of healthcare providers who fit the case criteria. Each of the four clinical institutions has demonstrated interest in genomic medicine by virtue of participating in National Human Genome Research Institute (NHGRI) consortiums, Implementing Genomics in Practice (IGNITE) coordinated at Duke University and the Electronic Medical Records and Genomics Network (eMERGE) coordinated at Vanderbilt University; however, they vary in the degree to which they have prioritized implementation of pharmacogenetics. For example, two institutions have policies to test preemptively patients for pharmacogenes and well- developed implementation processes, albeit at different stages, while others are at preparation or exploration stages of implementation (Table 1).

### 2.2. Data Collection

Data are collected via a structured questionnaire and semi-structured interview guide (see the appendix for questionnaire and interview questions). Taxonomies, which provide heuristics for describing empirical evidence by defining and categorizing constructs and common language for linking findings across different studies, guide data collection and analysis [22]. The web-based questionnaire, developed using Qualtrics, includes information about providers’ demographic and practice characteristics; intermediate outcomes of acceptability, appropriateness, and feasibility; and the outcome measure of pharmacogene test utilization. Semi-structured individual interviews with the same providers, conducted over Zoom, includes questions about factors hypothesized to impact the intermediate or final outcomes based on the Consolidated Framework for Implementation Research (CFIR), Theoretical Domains Framework (TDF), and Expert Recommendations for Implementing Change (ERIC) taxonomies (described in more detail below). Additional interviews will be conducted with one to two selected administrators at each health system to triangulate information on organizational-level factors.

### 2.3. Outcome Measures

The level of pharmacogenetic test utilization by providers who order antidepressant medications constitutes the primary outcome for the CNA, because the extent to which providers use this testing is the key factor in determining whether patients will ultimately reap the benefits. As an indicator of pharmacogene test utilization for prescribing antidepressants, we focus on tests that include the CYP2D6 or CYP2C19 genes for two reasons: (1) the CPIC provides evidence-based therapy recommendations for these genes depending on genetic test results and (2) providers across all institutions in our sample prescribe drugs that interact with these genes. We operationalize the level of utilization according to providers’ reports of the number of tests that they used in the past 6 months out of the number of patients they follow. We obtain this information by asking them during the pre-interview, Qualtrics questionnaire to report the number of tests that they ordered for CYP2D6 or CYP2C19 and then following up via the qualitative interview to ask about the number of tests that they used (e.g., discussed results with patients or used results to change medication). The way in which providers order CYP2D6 or CYP2C19 tests may vary- e.g., as a stand-alone test, within a panel of multiple PGx tests, or as part of an order set for a condition or diagnosis, and, while we collect this kind of process data during interviews, this detail does not affect the outcome definition. During the interview, we also ask about the number of patients they follow and what proportion of their patients are on tricyclic antidepressants or selective serotonin reuptake inhibitors that interact with CYPD26 or CYP2C19 (amitriptyline, citalopram, escitalopram, fluvoxamine, nortriptyline, paroxetine, or sertraline) to provide context for their pharmacogenetics test utilization numbers. We rely on self-reported data from providers to determine the extent to which they use pharmacogene tests for CYP2D6 or CYP2C19 within their own context, because systems to document orders of these specific gene tests for antidepressants vary across the clinical institutions. Using this self-reported information, we create outcome values by categorizing providers according to our final sample distribution (e.g., a trichotomous outcome of high, some, or no utilization categories or a dichotomous outcome of any or no utilization of tests).

### 2.4. Intermediate Outcomes

To identify the extent to which intermediate outcomes (antecedent outcomes that precede utilization) make a difference for the final outcome (level of utilization), we measure providers’ views on acceptability, appropriateness, and feasibility of pharmacogenetic testing as defined by the Implementation Outcomes Framework (IOF) [23] and using the validated Acceptability of Intervention Measure (AIM), Intervention Appropriateness Measure (IAM), and Feasibility of Intervention Measure (FIM) [24]. Acceptability generally refers to perceived satisfaction with a specific evidence-based practice, personal fit. Appropriateness refers to perceived fit of an evidence-based practice for a specific issue, relevant actors, and implementation settings. Feasibility refers to anticipated success of a new practice in a given setting. Each measure includes four items and evaluates the notion of fit. By measuring both acceptability and appropriateness, we tease out whether providers perceive pharmacogenetics as fitting for their overall practice setting (appropriate) though not desired by them in particular (acceptable), or vice versa.

### 2.5. Factors Hypothesized to Impact the Intermediate or Final Outcomes

(1) To identify multi-level determinants, defined as barriers and facilitators to implementation and practice improvement, we use the Consolidated Framework for Implementation Research (CFIR) and the Theoretical Domains Framework (TDF). The CFIR lists potential determinants selected from published literature based on strength of evidence or relevance for implementation and organized by five over-arching domains, or levels (intervention characteristics, individual characteristics, process, inner setting, outer setting) [25]. The TDF, developed from behavior change theory to identify how individual behavior influences implementation of evidence-based recommendations, is used to enhance the understanding of individual provider-level determinants for genomic medicine [26]. The TDF and CFIR together elucidate more organizational and individual-level determinants than either would alone. To our knowledge, this is the first pharmacogenetics implementation study to apply both CFIR and TDF to evaluate factors that make a difference for the level of provider utilization across different real-world institutional settings [27]. To narrow down the number of constructs to a manageable set for our qualitative and quantitative analyses, we use qualitative data to determine a set of constructs hypothesized to be most influential to level of utilization based on a series of discussions among the current study team about qualitative data analysis and then test the hypotheses using CNA.
(2) To identify whether and which implementation strategies make a difference in facilitating high versus low or no provider utilization, we use the Expert Recommendations for Implementing Change (ERIC) taxonomy, a compilation of 73 implementation strategies with definitions [28]. This taxonomy derives from both a systematic review of literature and examination by experts in health services research. To facilitate locating strategies, ERIC authors used concept mapping to divide the 73 strategies into nine clusters (engaging consumers, using evaluative and iterative strategies, changing infrastructure, adapting and tailoring to the context, developing stakeholder interrelationships, utilizing financial strategies, supporting clinicians, providing interactive assistance, and training and educating stakeholders). In our project, we use the ERIC taxonomy to deductively identify implementation strategies described in qualitative interviews, in response to questions about processes used to implement pharmacogenetic testing (e.g., What are steps to use pharmacogenetic tests in your health care system?).

Provider characteristics include number of years since completed training, provider type (MD or DO, PharmD or RPh, PA or NP, Other), primary clinical area (primary care, psychiatry, neurology, other), whether prescribing medications is part of job their responsibility, number of hours of direct patient care during a typical week, age, gender, race/ethnicity, primary location, and self-efficacy (confidence in ordering pharmacogenetic testing for CYP2C19 and CYP2D6 genes).

### 2.6. Analysis

#### 2.6.1. Phase 1: Qualitative Analysis

Analysts code segments of the transcribed interviews using descriptive labels as well as strength and valence codes (when applicable). Descriptive labels for coding text are created and defined using a combination of deductive and inductive approaches [29]. Deductive, a priori, codes are derived from CFIR and TDF factors. We establish additional inductive, or data-derived, codes for text that cannot be represented by a priori codes (e.g., apply codes that correspond to ERIC implementation strategies to text related to the question, “How do you integrate pharmacogenetics into your practice?” or apply codes for types of training described in response to the TDF skill question, “Have you been trained in how to use pharmacogenetics in your daily practice with patients? Please tell me more.”). To identify factors as facilitators or barriers, analysts assign numerical factor values (valence codes) to coded text to represent strength and valence (from −2 a strong barrier to +2 a strong facilitator) accompanied by rationale (e.g., provider expected to use pharmacogenetic testing and rewarded for doing so) in the annotation feature of NVivo, according to criteria developed during the review of data and based on guidance from prior studies that applied CFIR [30]. Analysts meet to review all coding with the principal investigator and resolve discrepancies in coding through discussion that includes additional team members with expertise in implementation science and pharmacogenetics implementation when needed. Discussion among all team members occurs to hypothesize which factors could make a difference for the outcome. A data matrix summarizes the coded data, with a row for each provider and values that were hypothesized to make a difference in separate columns. We resolve missing or unclear data by re-contacting respondents to check our interpretation or fill in their responses. The outcome is added as a final column and it is assigned a value of 0 for those providers with no test utilization, 1 for some and 2 for high utilization, or 0 for none and 1 for any utilization, depending on the sample distribution.

#### 2.6.2. Phase 2: Coincidence Analysis

CNA can uncover causal pathways by which factor values and implementation strategies (i.e., conditions) make a difference for level of provider utilization. CNA employs a mathematical algorithm based on Boolean algebra to compare case configurations and identify minimally necessary and sufficient conditions for a desired outcome. In this project, the outcome is denoted by extent of pharmacogenetic use (high or low) and the factors hypothesized to impact the outcome derived from prior qualitative analysis. Factors hypothesized to be most important to the outcome from team discussions during qualitative analysis are selected for inclusion in CNA to test the hypothesis. CNA methodology derives causal inference from empirical data through the modern Regularity Theory of Causation, which posits that if one event is regularly (consistently) followed by another in multiple instances (or cases), then causal inference can be made if redundancies are eliminated [31]. To interpret causal relationships identified in resultant solutions, we draw from implementation theory, qualitative data, and other knowledge (temporality, proximity).

One of the benefits of CNA is that it can uncover multiple paths to the same outcome [32]. In practice, this approach also allows for the identification of causal complexity, whereby two conditions if present alone are insufficient for the outcome, but together they may be minimally sufficient. For example, it may be that providers from academic centers with a longer-running program and strong positive implementation climate use clinical reminders to order tests (an implementation strategy), view pharmacogenetics as feasible (intermediate outcome), and, in turn, have higher rates of pharmacogene orders (final outcome). However, for providers in other health care settings, the “key ingredients” for achieving the outcome (high rates of ordering) may be the presence of high self-efficacy and use of local technical assistance (implementation strategy), which, in turn, results in views of pharmocogenetics as acceptable (intermediate outcome). In sum, CCMs provide a way to identify underlying complexity that can affect implementation in real world settings.

CNA is conducted using the “cna” package in R software [33]. We evaluate the fit of models generated by CNA using the standard parameters of consistency and coverage. Consistency refers to the proportion of cases with certain configurations of conditions in the solution that also has the outcome (number of cases with both the model solution and outcome divided by the number of cases with the model solution). Coverage refers to the degree to which a specific model solution accounts for the behavior of an outcome and is measured by the number of cases with both the model solution and outcome divided by the total number of cases with the outcome). Consistency and coverage are initially set at 85 to guard against model overfitting. Robustness testing is conducted using the R package “frscore,” which computes the degree to which a model fits with other models generated from the same dataset under varying thresholds of consistency and coverage [34]. If CNA uncovers model ambiguity, that is, no one stand out model is identified, the models are evaluated based on coverage, consistency, and fit robustness scores to determine the best fitting and most robust model.

## 3. Discussion

Context matters for implementation of any evidence-based practice and may have unique implications for precision medicine. Translating an evidence-based practice, like pharmacogenetic testing, into different settings opens the door to changing components of the practice and maximizing beneficial outcomes. Understanding the role of context, or surrounding conditions and implementation strategies, can help to identify why a practice that works well in one setting may not in another, how to improve fit without compromising outcomes, and areas to intervene to improve implementation. While implementation science frameworks have generally focused on external features like organizational culture or implementation climate to define context, individual-level factors like end-users’ knowledge or beliefs also can affect how a new practice fits in a specific setting. This multi-level view of context matters for precision medicine applications that depend on use of clinical decision support systems that process big data sources to interpret results for just-in-time guidance in varying clinical contexts: For example, the extent to which providers have knowledge necessary to use a clinical decision support system may shape implementation climate or overall receptivity.

Because intervention effectiveness depends on multi-level contextual factors, we need ways to measure them to evaluate interventions. Rogers and colleagues (2020) conducted a systematic review to assess how studies of healthcare implementation defined and measured “context” and found that the majority of studies used only qualitative methods and those that used quantitative methods relied on cross-sectional surveys; generally, these studies generated listings of relevant factors from frameworks to define the notion of context [35]. CCMs add to the toolbox of methods for measuring contextual factors as multi-level, multi-component conditions for implementation; an advantage of CNA is that it offers flexibility to uncover contingencies, or combinations of conditions, as pathways [30]. In this way, CCMs in general and CNA in particular fit well with the idea of context as process, in which dynamic conditions intersect with features of the intervention and other factors (e.g., individual characteristics), as opposed to a fixed structure to control for as a potential confounder [36]. There is growing consensus around use of CCMs in implementation science and their importance in studying the implementation of precision medicine [37]. Currently few methodologies exist to study how contextual factors affect implementation and generate causal models: There needs to be greater use of innovative methods to answer these kinds of questions pertinent for implementation contexts [38].

For precision medicine specifically, the study of implementation into routine care is needed for continual development of evidence about clinical utility. This represents a virtuous cycle between research and implementation, where basic science can gain from insights generated by learning health care systems and vice versa [39]. By studying implementation, we can fully identify barriers and concerns around provider use of pharmacogenetics, which will help sites that are beginning to implement. For example, knowing what specifically discourages providers from utilizing and ordering tests will be helpful to more effectively address these concerns through communication strategies. Providing a systematic way to generalize across institutional settings with common language and definitions provided by implementation science frameworks helps to build general knowledge. While this work extends current approaches to elicit barriers through surveys, interviews, and observations, it still represents only a piece of the puzzle. Future work to study difference makers for uptake of pharmacogenetics must include patient experiences. While we do not focus on patient-level analyses for the study described in this paper, a separate qualitative study will collect information from patients using the same implementation science frameworks to generate hypotheses and test the hypotheses using CCMs. In general, the study of precision medicine implementation can benefit from using CCMs to understand how complex, real-world conditions affect uptake and highlight areas to intervene to improve outcomes.

## Figures and Tables

**Table 1:** Description of clinical institutions.

Clinical Institution	Patient Mix	Health Care Setting	NIH Clinical Genomics Network	Program Stage ^1,2^
Duke Health (Durham, NC, USA)	55% White; 20% Black; 5% Hispanic14% Medicaid	Urban academic medical center	IGNITE	Exploration
Geisinger Clinic (Danville, PA, USA)	90% White; 32% Geisinger Health Plan membership covered by Medicaid	Regional system reaching rural areas	eMERGE	Preparation
Cincinnati Children’s Hospital (OH, USA)	75% White; 15% Black; 8% Hispanic43% Medicaid	Urban academic medical center	IGNITE affiliate; eMERGE	Implementation (preemptive)
Sanford Health (Sioux Falls, SD, USA)	85% White; 4% Black; 9% Native American (wide variations among regional facilities). 12% Medicaid	Regional system reaching rural areas	IGNITE affiliate	Expansion(^3^ preemptive and reactive)

Note: ^1^ Smith, B., Hurth, J., Pletcher, L., Shaw, E., Whaley, K., Peters, M., & Dunlap, G. A guide to the implementation process: stages, steps and activities. Chapel Hill: The University of North Carolina, Frank Porter Graham Child Development Institute, The Early Childhood Technical Assistance Center **2014**; ^2^ Aarons, G. A., Hurlburt, M., & Horwitz, S. M. Advancing a conceptual model of evidence-based practice implementation in public service sectors *Administration and Policy in Mental Health and Mental Health Services Research*, **2011**, *38*(1), 4–23; ^3^ preemptive = tests conducted prior to drug prescribing and readily available in patient medical record, and reactive = test conducted at time of prescribing, Weitzel, K. W., Cavallari L.H. and Lesko L.J. Preemptive Panel-Based Pharmacogenetic Testing: The Time is Now. *Pharmaceutical Research*, **2017**, *34*(8), 1551–1555.

## Data Availability

Not applicable.
